# Using population isolates in genetic association studies

**DOI:** 10.1093/bfgp/elu022

**Published:** 2014-07-09

**Authors:** Konstantinos Hatzikotoulas, Arthur Gilly, Eleftheria Zeggini

**Keywords:** isolated populations, rare variants, complex disease, genetic association studies

## Abstract

The use of genetically isolated populations can empower next-generation association studies. In this review, we discuss the advantages of this approach and review study design and analytical considerations of genetic association studies focusing on isolates. We cite successful examples of using population isolates in association studies and outline potential ways forward.

Genome-wide association studies (GWAS) have met with widespread success in identifying common-frequency variants associated with complex diseases and medically relevant quantitative traits. Technological advances in genotyping and sequencing have recently enabled access to low-frequency and rare variant genotypes at the population level. The identification of modest effects at individual low frequency and rare variants requires very large sample sizes. Power can be boosted by using statistical approaches to aggregate rare variants across chromosomal regions or functional units. Power can also be increased by leveraging the unique characteristics of isolated, or founder, populations in genetic association studies. In the past, population isolates have typically been used in family-based genetic studies of Mendelian traits [1, 2]. Advances in genotyping and sequencing technologies have catalysed the use of founder populations in genetic association studies of complex traits. Here, we review the population genetics characteristics of isolated populations, outline study design and analytical considerations and discuss examples of next-generation association studies in population isolates.

## PROPERTIES OF POPULATION ISOLATES

**Figure 1:**
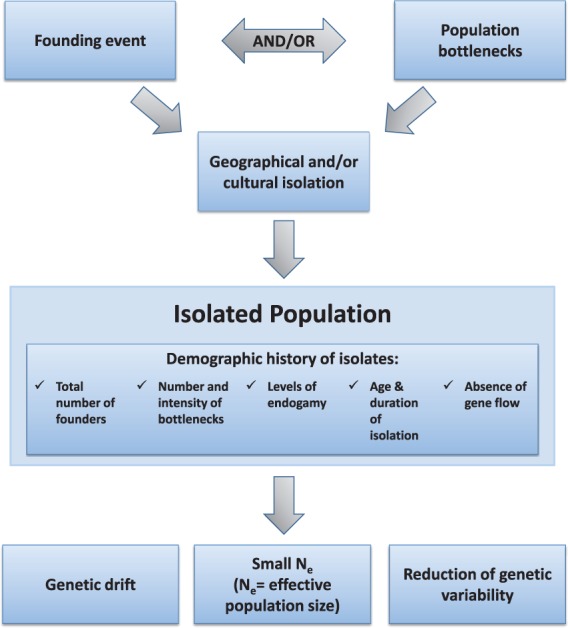
Factors that shape population isolate characteristics.

Population isolates can be defined as subpopulations derived from a small number of individuals who became isolated because of a founding event (e.g. settlement of a new territory, famine, war, environmental disruption, infectious disease epidemics, social and/or cultural barriers) and have stayed so for many generations. The resulting geographical and/or cultural isolation of these populations has genetic consequences, as, for example, endogamy (within community marriage) along with very restricted gene flow (immigration) from neighbouring populations can often be observed. Thus, genomes tend to show higher homogeneity in isolates compared with cosmopolitan populations, which is reflected by a reduced effective population size (N_e_ or the effective number of individuals required to explain the observed genetic variability) [3] (Figure 1).

Another potentially advantageous property of population isolates is environmental and cultural homogeneity. In addition to reduced genetic complexity, individuals from an isolated population tend to share a common lifestyle, including diet, physical activity levels and other cultural habits, and, importantly, are exposed to similar environmental and sanitary conditions and disease vectors. Phenotype definition and diagnosis harmonization can also be achieved through standardized clinician training, a model adopted, for example, by Finland.

Isolates can branch and develop independently; geographical proximity and common origins do not imply identical evolution. Many isolates experience more than one founding event, which can result in population bottlenecks [4] or the creation of regional subisolates [5]. A typical example is Finland, where both older (∼2000 years) and younger (∼500 years) population isolates have appeared within one geographical region due to internal migration [6]. Various factors such as the total number of founders, number and intensity of bottlenecks, levels of endogamy, age and duration of isolation shape the demographic history of isolates (Figure 1). Detailed genealogical records are sometimes available for isolated populations. For example, the Icelandic population has an extensive genealogical and disease history database [7, 8]. Given genome-wide data availability, the individual characteristics of isolates can be examined using population genetics analysis tools [9–12].

## GENETIC CONSEQUENCES OF ISOLATION

### Reduced haplotype complexity

Thanks to the HapMap [13] and 1000 Genomes Projects [14], we now have a good understanding of linkage disequilibrium (LD) patterns across the human genome for several human ethnic groups. In isolated populations, LD tends to extend over longer distances compared with non-isolated populations, as exemplified by studies in the populations of the Central Valley of Costa Rica [15], Palau [16] and Val Borbera in northwest Italy [9]. As expected through ancestral recombination, the LD intervals of older isolates tend to be shorter than those of younger isolates [17]. Moreover, relatively higher levels of LD are observed in isolates with a small number of founders that experience slow growth during the early generations following the initial bottleneck [18]. Longer stretches of LD in isolates mean longer haplotypes, thus facilitating disease association studies and empowering imputation approaches that infer genotypes at untyped variants based on regional LD information [19]. The disadvantage of high levels of correlation among sequence variants is a reduction in resolution of the localization of causal variants within a wide association peak signal. Trans-ethnic meta-analysis has been proposed as an approach to synthesize data across populations with diverse LD patterns to enable fine-mapping of the causal variant(s) [20].

### Reduced allelic variability and genetic drift

Isolation can influence the patterns and prevalence of disease. Notably, owing to the enrichment of some rare alleles resulting from the combined effect of endogamy, bottlenecks, genetic drift, recurrent mutation and selection, isolates have been shown to potentially exhibit an increased incidence of recessive disorders. Each isolate shows a unique profile of rare disease alleles [21], which can be expressed through a higher prevalence of some diseases and lower incidence of others [22, 23]. For example, the Pima Indians of Arizona have a very high prevalence of type 2 diabetes (∼20%) [24, 25] and near absence of type 1 diabetes [26].

In population isolates, certain alleles reach fixation or extinction at a particular locus, thus reducing the extent of genetic variability [27, 28]. Some variants that contribute to complex traits/diseases are rare in the parent population and drifted to higher frequency in the isolate. The enrichment of low-frequency alleles in the study population can empower the identification of these variants with smaller discovery sets. For example, a null mutation in *APOC3* that had risen in frequency in the Amish founder population was found to be associated with a favourable plasma lipid profile. Association with this variant (previously thought to be private to the Amish) was recently replicated in an exome chip-wide association scan for lipid traits in ∼1200 individuals from a Greek population isolate [29]. To achieve 80% power to detect the observed effect size in the general European population (in which the variant has 40-fold lower frequency), a sample size of 67 000 individuals would have been required. The phenomenon of reduced allelic variability, combined with extended LD, is expected to improve power for trait association at rare variants compared with populations with wider allelic diversity (noting that other rare variants will be lost).

## STUDY DESIGN

Population choice is an important consideration in designing a genetic association study focusing on isolates. Factors such as the number of founding haplotypes, age of divergence from the parent population, effective sample size and degree of admixture with neighbouring populations, all play a role in the population’s allelic architecture. For initial GWAS as well as rare Mendelian gene discovery, the study of young founder populations with recent expansion (e.g. late-settlement Finland) is a powerful strategy [30] because of their higher degree of LD and reduced genetic diversity. It has been suggested that small populations that have remained stable throughout most of their history could lead to more economical locus discovery efforts [31]. Drift of rare alleles occurs at random and for a small set of variant sites; therefore, the power of a genetic association study in a population isolate will depend on the enrichment of alleles that are relevant to a phenotype of interest [32–34], which requires the alleles in question to have passed through the initial population bottleneck. An association study in an isolate can often be motivated by a suspected higher prevalence of a trait or disease in that particular population.

## TO TYPE OR TO SEQUENCE?

GWAS arrays by definition assay primarily variants selected to represent common frequency variation across the genome. Low-frequency and rare variants genome-wide cannot be easily captured on a genotyping array because of their large numbers and low levels of correlation. Recently, a genotyping chip focused on likely functional coding low-frequency and rare variants—the exome chip—has been used successfully in founder populations to associate rare variants with proinsulin [35] and HDL cholesterol [29] levels. The decreasing cost of sequencing makes it increasingly easier to study the complete variation landscape irrespective of allele frequency [36].

Whole exome sequencing has the advantage of reduced cost compared with whole-genome sequencing, but does not capture variation in non-genic regions. Previous experience from GWAS strongly indicates that the majority of complex trait signals reside outside of genes. High-depth sequencing is considered necessary to call high-quality variants across the allele frequency spectrum [37]. However, it has been shown that in the context of a population study, whole-genome sequencing many individuals at low depth can have variant detection power advantages over fewer individuals sequenced at higher depth [38, 39]. This approach was tested by the 1000 Genomes Project pilot and has been used to generate the widely used phase 1 and phase 2 variant sets.

When not all samples can be sequenced, whole-genome sequencing of a subset of cases and controls following an initial GWAS has proven to be a successful strategy for empowering rare variant association in dichotomous trait studies [40, 41]. Variants from the sequenced samples are phased using long-range haplotype phasing (LRP; see Imputation), then imputed back into the whole sample set, which is equivalent to using the sequenced subset as a reference panel for imputation.

## ANALYTICAL CONSIDERATIONS

### Relatedness

One intrinsic consequence of genetic isolation is relatedness among individuals, which can conflict with the assumptions of independence of many commonly used analysis tools and inflate test statistics affecting association signals. An efficient approach is to account for relatedness in the association analysis through the use of a linear mixed model (LMM). Until recently, computation of an exact association test statistic such as the Wald statistic or likelihood ratio (implemented in EMMA [42]) was computationally impractical. Tools that compute approximate solutions, either by using the residuals from the LMM under the null model as phenotypes, such as GenABEL [43], or by avoiding the repeated estimation of variance components, such as TASSEL [44] or EMMAX [45], have recently been developed. Mathematically optimized versions of the exact test, such as GEMMA [46] or FaST-LMM [47], have also been developed and are widely used.

Several methods have been proposed to improve on the power of single-point tests for rare variants by combining information across multiple sites in a chromosomal region and testing for association with the trait of interest [48–51]. Relatedness information can be incorporated in the model, such as in famSKAT [52] or other tools [53–57].

### Imputation

When performing association based on genotyping arrays, it is common practice to impute untyped variants based on a reference panel (e.g. the 1000 Genomes Project (www.1000genomes.org) and/or the UK10K study data (www.uk10k.org) to enhance the resolution of the study. This approach, where positions that were not genotyped in the sample are added using phase information in the reference set, is also relevant to refining genotype calls for low-depth sequencing data.

Imputation is closely related to phasing, a procedure that infers haplotypes based on identity by state (IBS), with other phased individuals. Relatedness is helpful for phasing because it increases the likelihood of finding a long IBS string of variants; the more related the samples, the more certain it is that these IBS sequences are actually inherited identical-by-descent (IBD), and the probability that unobserved positions are IBS becomes quantifiable [58, 59]. Kong et al. [60] proposed LRP, a method that uses regions of IBD between related individuals within the sample, to phase and impute variants. This approach has been generalized and improved to achieve higher accuracy around recombination sites in e.g. SLRP [61], ANCHAP [62] and other methods [63].

### Meta-analysis

The synthesis of data through meta-analysis can increase the power of association studies. Two different classes of methods have been typically applied in traditional meta-analysis of GWAS: *P*-value-based tests and effect size-based methods, which can be further subdivided into fixed or random effects models [64]. Fixed effects models assume that the same underlying effect is present in all studies, whereas random effects models allow for effect sizes to be different. These approaches can be applied to meta-analysing data across isolates. However, in the era of rare variant association testing, allelic heterogeneity can decrease power either because of the presence of similarly associated multiple rare variants or different ethnic backgrounds in the populations being meta-analyzed [65]. In addition, meta-analysis generally assumes independence of the study samples, which does not hold in the case of within-isolate meta-analysis. Research in this field is still ongoing [20], and a continued effort in method development is needed.

## COMPLEX TRAIT LOCUS DETECTION IN FOUNDER POPULATIONS

Several studies from Iceland have been successful in identifying low- /rare-frequency variants associated with sick sinus syndrome, gout, prostate cancer and Alzheimer’s disease [41, 66–68]. In a recent study in Finland, four novel loci were found to be associated with saccular intracranial aneurysms, a complex trait with a sporadic and a familial form [69]. One of these variants has drifted up 15 times in frequency compared with the Dutch general population and is virtually non-existent in other populations from the 1000 Genomes Project. A genome-wide significant risk locus for schizophrenia and bipolar disorder has been identified in an ethnically homogeneous cohort of Ashkenazi Jewish individuals [70]. The top signal (rs 11098403) is an inter-genic variant located in *NDST3* and was replicated in 11 independent cohorts of varying ethnicities. Recently, a Greek isolated population replicated a genome-wide significant association between R19X, a cardioprotective variant in *APOC3*, and low blood triglyceride levels [29]. This study also demonstrated that associations discovered in population isolates can be generalizable, as the same variant (R19X) had previously been discovered in the Amish founder population [71].

## FUTURE DIRECTIONS

Population isolates are uniquely positioned to usher in the new era of sequence-based association studies. The relative power advantages afforded by studying isolates have been well-documented and recently exemplified in the literature through successful identification of complex trait loci that replicate in other populations. Not all novel discoveries in isolates can be recapitulated in other populations. However, private variants detected in isolates can importantly point to novel biology, identifying potential pathways and loci involved in the aetiopathogenesis of clinically relevant traits. Large-scale efforts to synthesize genome-wide data across isolated populations are an intuitive next step in the field, and hold the promise of catalysing the discovery of further complex trait-associated variants.

**Key points**

- The population genetics characteristics of an isolate depend on demographic history and the number of bottlenecks/founding events, total number of founders, levels of endogamy, age and duration of isolation.
- Genetic consequences of isolation such as reduced haplotype diversity and genetic drift can enhance the power for locus identification in genetic association studies of complex traits.

## FUNDING

This work is supported by the Wellcome Trust (098051) and the European Research Council (ERC-2011-StG 280559-SEPI).
